# Impact of the Invasive Argentine Ant in Citrus Agroecosystems: Effects on the Diversity and Frequency of Native Ant Species Foraging on Tree Canopy

**DOI:** 10.3390/insects11110785

**Published:** 2020-11-11

**Authors:** Vera Zina, Manuela Branco, José Carlos Franco

**Affiliations:** 1 Centro de Estudos Florestais, Instituto Superior de Agronomia, Universidade de Lisboa, Tapada da Ajuda, 1349-017 Lisbon, Portugal; mrbranco@isa.ulisboa.pt (M.B.); jsantossilva@isa.ulisboa.pt (J.C.F.); 2 Departamento dos Recursos Naturais, Ambiente e Território, Instituto Superior de Agronomia, Universidade de Lisboa, Tapada da Ajuda, 1349-017 Lisbon, Portugal; 3 Departamento de Ciências e Engenharia de Biossistemas, Instituto Superior de Agronomia, Universidade de Lisboa, Tapada da Ajuda, 1349-017 Lisbon, Portugal

**Keywords:** ants, Formicidae, invasive species, *Lasius grandis*, *Linepithema humile*, principal response curve, seasonal changes

## Abstract

**Simple Summary:**

We aimed at assessing the impact of the Argentine ant invasion on the native ant community in citrus ecosystems. We compared the Argentine ant’s invaded and uninvaded citrus orchards in the south of Portugal, estimating species richness and the frequency of ant assemblages foraging on the tree canopy. The results suggest that the Argentine ant has a negative impact on the native ant community structure, markedly reducing the diversity and frequency of native species. This impact was more or less pronounced depending on the season. Possible implications for citrus pest management are discussed.

**Abstract:**

The invasion of the Argentine ant, *Linepithema humile* (Mayr) (Hymenoptera, Formicidae) can alter the entire ecosystem with serious impacts on the native community structure (e.g., ant diversity) and processes (e.g., trophic interactions) leading to biodiversity loss and pest outbreaks. Most studies addressing these impacts have been conducted in natural or semi-natural areas, few are those conducted in agricultural ecosystems, such as citrus orchards. These are dominant agricultural ecosystems in Mediterranean landscapes. Furthermore, most studies have been conducted in a short span, not evidencing seasonal fluctuations. In this work, we assessed the ecological impact of the Argentine ant on the native ant communities in citrus orchards, in the region of Algarve, southern Portugal. By using principal response curve, we compared seasonal variation on ant assemblages in invaded and uninvaded citrus orchards foraging on tree canopy from a two-year sampling. The Argentine ant had a marked negative impact on the native ant community foraging on citrus canopy. In the uninvaded orchards, the native ant community had a rich assemblage composed of 16 ant species, in its majority (72%) controlled by the dominant species *Lasius grandis* Forel, *Tapinoma nigerrimum* (Nylander) and/or *Pheidole pallidula* (Nylander). In the invaded orchards, the native ant community was poorer and highly modified, mostly dominated by the Argentine ant (80%). Apparently, the only native ant species not affected by the presence of the Argentine ant was *Plagiolepis pygmaea* (Latreille). A significant negative effect was found between the proportion of infested trees by *L. humile* and the number of native ant species per orchard. Differences in the native ant community in the invaded and uninvaded orchards persisted over seasons and years. However, negative impacts were higher in the spring and summer, and less pronounced in the autumn. We discuss implications for citrus pest management.

## 1. Introduction

Ant invasions represent a worldwide concern and have been the subject of the largest number of publications on invasive insect studies in recent years [1]. Most of the introduced ant species are usually not detected and usually do not constitute a threat to native fauna. However, those species that become invasive are very successful and considered a serious threat to the world’s native biodiversity [2,3]. The causes underlying the ecological success of invasive ants have been documented [4]. Apparently, it seems that a combination of characteristics such as omnivory, unicoloniality, absence of competitors and natural enemies makes them successful invaders [4]. The Argentine ant, *Linepithema humile* Mayr, appears to have these abilities [4]. Native to South America, this species easily spread and become established into new areas around the world [5]. It has been recognized that the Argentine ant can displace the native ant community leading to dramatic impacts on the ecosystems [4,6,7,8,9,10]. 

Understanding the ecological impacts of invaders and the mechanisms underlying invasion dynamics is of extreme importance to anticipate or mitigate their negative environmental impacts [11]. However, there are difficulties in performing experimental studies on the impact of invasive ants since researchers cannot ethically introduce invaders to uninvaded areas [12]. As such, the impacts of invaders have been quantified by comparing selected metrics between invaded and uninvaded areas [13,14,15,16]. Such comparisons can yield important insights into a wide variety of impacts associated with ant invasion [4]. 

To date, most studies on the impact of invasive ants have been conducted in natural or semi-natural areas [4,6,17]. Only a few were conducted in agricultural ecosystems, such as citrus orchards [18]. 

Citrus is an economically important irrigated crop in the Mediterranean region, representing 20% of the world’s citrus production [19]. Ants are common insects foraging on citrus canopy [20,21], mainly due to the high diversity of honeydew-producing hemipteran in this crop, including aphids (Hemiptera, Aphidoidea), whiteflies (Hemiptera, Aleyrodoidea), and scale insects (Hemiptera, Coccoidea) [22,23,24,25,26,27,28,29]. The Argentine ant reaches high densities in Mediterranean citrus orchards, where it is considered a common pest, also facilitating the activity of some sap-sucking insect species [30].

The decreasing diversity and abundance of native ants resulting from ant invasions can give rise to a variety of direct and indirect effects on non-ant taxa [4]. This is of extremely high importance for integrated pest management (IPM), as the composition of ant communities may influence the pest status of insect species present in agricultural ecosystems. For example, the most recent update to the USA Road Map for IPM places a special emphasis on invasive species [31].

In a previous study on ant communities associated with citrus, in the region of Algarve (south of Portugal), the Argentine ant was detected in 33% of the sampled orchards within two subregions, and was completely absent from another subregion—Serra [21]. Since Algarve was the first Mediterranean area invaded by the Argentine ant, over 120 years ago [32,33] it is likely there was no limitation of time for its establishment in the region. Based on this assumption, Zina et al. [21] hypothesized that, among other factors, the absence of the Argentine ant in the Serra could be explained as the result of interspecific competition with dominant native ant species, such as *Lasius grandis* Forel, *Pheidole pallidula* (Nylander) and *Tapinoma nigerrimum* (Nylander). 

In the present study, we aimed at assessing the impact of the Argentine ant invasion on the native ant community foraging on the tree canopy in citrus ecosystems. As an experimental approach, we compared the species richness and frequency of ant assemblages foraging on the tree canopy between invaded (treatment) and uninvaded (control) citrus orchards in the region of Algarve. It was assumed that differences between treatments on the structure of ant communities is a result of the interaction between native ants and the Argentine ant. We further hypothesized that those interspecific interactions changed over the year. Autumn and winter must be a particular moment in which the Argentine ant is most vulnerable, as this species is known to have seasonal polydomy, a type of intrinsic nest relocation pattern in which colonies converge during the winter and spread among multiple nests in warmer periods [34,35,36]. Overall, we predict finding a higher number of ant species in uninvaded than invaded orchards, as well as a seasonal effect on this difference in species richness. This study was thus conducted during two years, with seasonal sampling, in order to identify the possible existence of seasonal patterns in ant species diversity and frequency. 

Experimental studies and research focusing on the invasive ecology of ants and impacts on the native ant community are especially valuable contributions to agrobiodiversity and citrus pest management. Thus, we discuss the possible implications of the results for citrus pest management. 

## 2. Materials and Methods

### 2.1. Study Area and Experimental Design

This study was conducted in Algarve, the main citrus producing region of Portugal. With a Mediterranean climate, the average annual temperature in the region is 15.5–17.5 °C, decreasing to 13 °C at 900 m altitudes. The maximal temperature in the summer can reach 30–35 °C, occasionally 40 °C [37]. 

**Figure 1:**
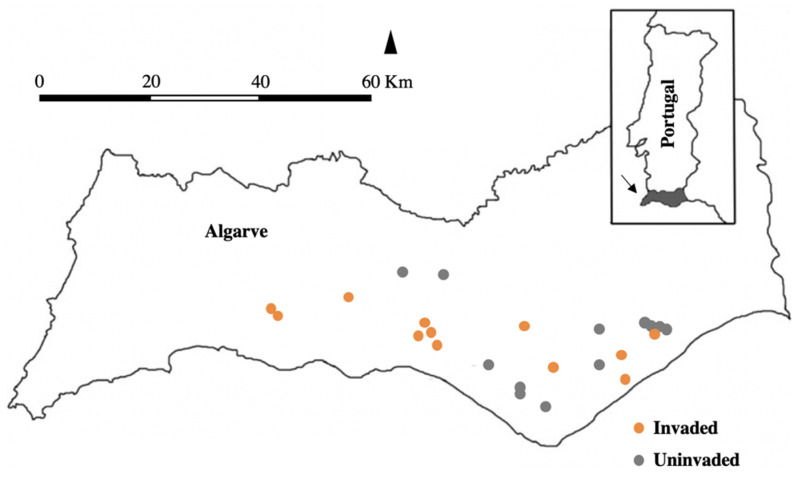
Map of the Algarve region with the invasion status of sampled orchards. The orange dots represent the invaded orchards by the Argentine ant, while grey dots represent uninvaded orchards.

Based on a previous study [21], we selected 24 commercial citrus orchards, distributed between Silves (37.177 N, 8.448 W) and Tavira (37.154 N, 7.652 W) (Figure 1), 12 of which were invaded by the Argentine ant. The orchards included 71% of sweet oranges, *Citrus sinensis* (L.) (cv. Valencia Late, Newhall, Navelina, Rhode, Navelate Lanelate, Salustiana), 13% lemons, *C. limon* Osbeck (cv. Eureka), 8% mandarins, *C. reticulata* (Blanco) (cv. Ortanique, Encore), and 8% mixed plots, with sweet oranges and mandarins. The average area per citrus plot was identical (*p* = 0.57) between invaded (1.46 ha ± 1.08 SE) and uninvaded (1.73 ha ± 1.25 SE) orchards. The nearest neighbour index among orchards was 0.88 (Z-Score = −1.14). Eventually, insecticide treatments under an IPM regime were applied to control key pests, such as the California red scale, *Aonidiella aurantii* (Maskell) and the Mediterranean fruit fly, *Ceratitis capitata* (Wiedemann). We assumed no differential effects of insecticide treatments between invaded and uninvaded orchards. Experimental evidence supports this assumption, as ant community structure has been shown to be insensitive to pesticide gradients (e.g., [38]).

### 2.2. Ant Sampling and Identification

Seasonal samplings (summer, autumn, spring) were carried out during two years, between July 2012 and May 2014, in the same 20 trees per orchard. Trees were first selected at haphazardly along 4–5 line transects, each tree separated from the other by at least 15 m to avoid spatial correlation. Line transects covered the central part of the studied citrus plots, as a grid, in order to consider within orchard variability. Samplings were carried out between 17 July and 24 August, 17 October and 15 November, in 2012; between 25 March and 26 May, 18 July and 23 August, 15 November and 30 November, in 2013; and between 19 May and 28 May, in 2014. For each sampling period, the number of sampled plots in each date was similar between invaded and uninvaded orchards. The method used was direct search by hand collection, considered the most efficient technique to estimate the diversity of ant species [39,40]. This method has proved effective in other ant studies conducted in Mediterranean citrus groves [20,21,41]. It records the presence of species inhabiting a habitat element and allows listing the ant fauna in relatively short time by experienced collectors [42]. The presence of ants in the tree canopy was determined by visual observation of the trunk, shoots, leaves, fruits and flowers, along the tree canopy perimeter up to 1.70 m height, during 60 s per tree, following Zina et al. [21]. This time is sufficient to determine the presence of ants in a tree by ant experts. Whenever ants were detected, a sample of specimens was collected and preserved in 96% alcohol, within Eppendorf tubes, for species identification. The samples were stored in the Entomology Laboratory of the School of Agriculture, University of Lisbon. The collected ant specimens were observed under magnification (640×) and identified at the species level based on Collingwood and Prince [43] and Gómez and Espadaler [44].

### 2.3. Data Analysis

We built a generalized linear mixed model (GLMM) with the Poisson distribution using a log link function to explain observed variation in ant diversity. We used the orchards as subjects with repeated measures, the year was considered a random variable, and the season and the treatment as fixed factors, with three and two levels, respectively. Pairwise comparisons of estimated marginal means were used to estimate significant differences in ant species richness between invaded and uninvaded orchards, and among seasons. The GLMM was fitted using SPSS [45].

Pearson’s correlation tests were performed by season and overall to assess the relationship between the proportion of infested trees per orchard and the number of native ant species in those orchards.

Multivariate statistical analysis was used to describe the effects of Argentine ant’s invasion at the community level. The principal response curve (PRC) method was applied to study the effect of the invasion by the Argentine ant on the native ant community, by comparing the native ant species over the sampling seasons in invaded and uninvaded orchards, using packages Vegan for R version 3.3.1 software for Windows [46], with data on occurrences per orchard. Monte Carlo permutation tests [47] were performed to test the significance of the first axis and the significance of the PRC deviations for each sampling season. PRC is based on redundancy analysis (RDA), adjusted for overall changes in community response over time, in relation to the control [48]. This is an interpretive method allowing a quantitative interpretation of the effects headed for the species level enabled by scoring the species weight, accounting for deviances [47].

## 3. Results

### 3.1. Ant Communities

**Figure 2:**
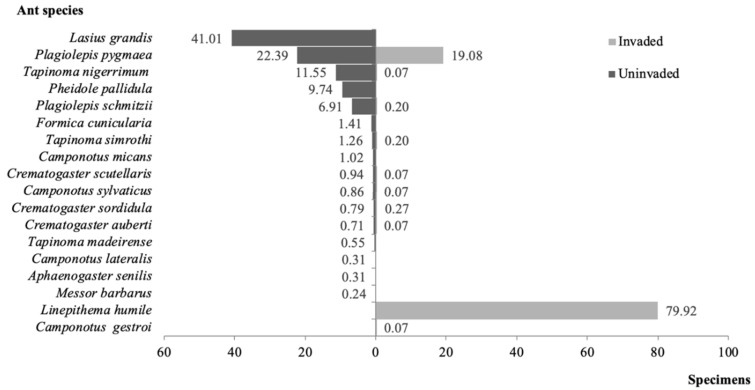
Frequency distribution (%) of ant species in Argentine ant invaded and uninvaded orchards.

In total, 10,930 individuals comprising 18 ant species, 10 genera, and three subfamilies were collected in the sampled orchards (Table 1). Of these, 55% were collected in the Argentine ant’s invaded orchards, and 45% in the uninvaded orchards (Table 1). Overall, ants were present in all sampled orchards and in 81% of the sampled trees (89% and 72%, in invaded and uninvaded orchards, respectively). Sixteen ant species were identified in uninvaded orchards, while only 10 species were observed in the orchards invaded by the Argentine ant. In the first case, five ant species, i.e., *L. grandis* (41% of the specimens), *Plagiolepis pygmaea* (Latreille) (22%), *T. nigerrimum* (12%), *P. pallidula* (10%), and *P. schmitzii* Forel (7%) represented 92% of the collected specimens, whereas in the case of invaded orchards, 99% of the specimens belonged to *L. humile* (80%) and *P. pygmaea* (19%) (Figure 2). 

A significant negative correlation (at the 0.05 level) was found between the magnitude of invasion by *L. humile* (i.e., the proportion of infested trees) and the number of native ant species per orchard (Pearson Correlation (PC) = −0.42, *p* < 0.001; Appendix A). Individual correlations by season showed the same trend for summer (PC = −0.47, *p* = 0.019; Appendix A) and spring (PC = −0.46, *p* = 0.026; Appendix A), but not for autumn (PC = −0.17, *p* = 0.425; Appendix A). When *L. humile* was present, native ants were found only in very low levels (up to nine individuals). Most species were found together with *L. humile* in just one orchard (*Crematogaster scutellaris* (Olivier), *C. sordidula* (Nylander), *C. auberti* Emery, *Camponotus sylvaticus* (Olivier), *C. gestroi* Emery, *T. nigerrimum*, and *T. simrothi* Krausse). *Plagiolepis schmitzii* co-occurred with the Argentine ant in two orchards. An exception was found for *P. pygmaea*, which was observed in all invaded orchards (Figure 2, Table 1). Eight ant species, i.e., *Aphaenogaster senilis* Mayr, *C. lateralis* (Olivier), *C. micans* (Nylander), *Formica cunicularia* Latreille, *L. grandis*, *Messor barbarus* (Linnaeus), *P. pallidula*, and *T. madeirense* Forel appeared only in uninvaded orchards, whereas *C. gestroi* was only observed in an invaded orchard (Figure 2, Table 1). 

**Figure 3:**
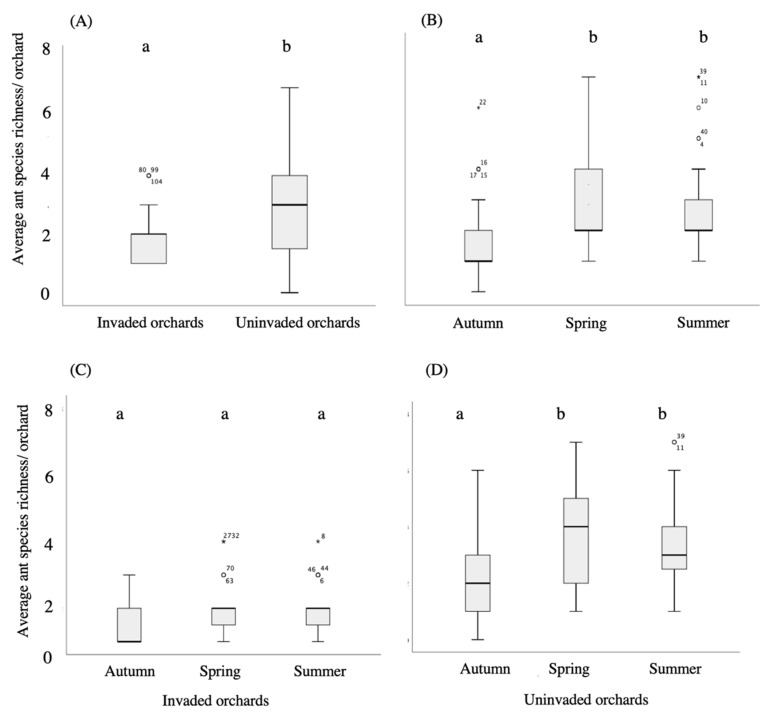
Box plots representing the average ant species’ richness per orchard in function of the invasion of the Argentine ant (**A**) and the season (**B**) in invaded (**C**) and uninvaded (**D**) citrus orchards. Boxes show interquartile ranges (25th and 75th percentiles), middle lines are medians, whiskers are non-outlier ranges beyond the boxes, circles are the outliers and asterisks are the extreme outliers. Different letters show significant differences between invasion treatment groups and among seasons by the fitted generalized linear mixed model (GLMM).

Overall, the results on the GLMM showed that ant species richness is significantly affected by treatment (F_(1,138)_ = 17.90, *p* < 0.001) (Appendix B). The average number of ant species per orchard in uninvaded orchards (2.98 ± 0.23; maximum = 7) was significantly (*p* < 0.001) higher than in invaded ones (1.77 ± 0.17; maximum = 4) (Figure 3A). We present the analyses of all pairwise comparisons in Appendix C.

### 3.2. Seasonal Patterns

Ants were observed in all seasons and orchards, except for autumn 2012 and 2013, in which ants were observed in about 92% (11 out of 12) and 83% (10 out of 12) of the invaded and uninvaded orchards, respectively. Overall, the results on the GLMM showed that ant species richness is significantly affected by the season (F_(2,138)_ = 5.50, *p* = 0.005) (Appendix B). The average number of ant species per orchard was significantly lower in autumn (1.73 ± 0.17) than in spring (2.69 ± 0.25; *p* = 0.002) and summer (2.60 ± 0.25; *p* = 0.005) (Figure 3B). No significant differences were found between spring and summer (*p* = 0.80), both in invaded (Figure 3C) and uninvaded orchards (Figure 3D). We present the analyses of all pairwise comparisons in Appendix C.

**Figure 4:**
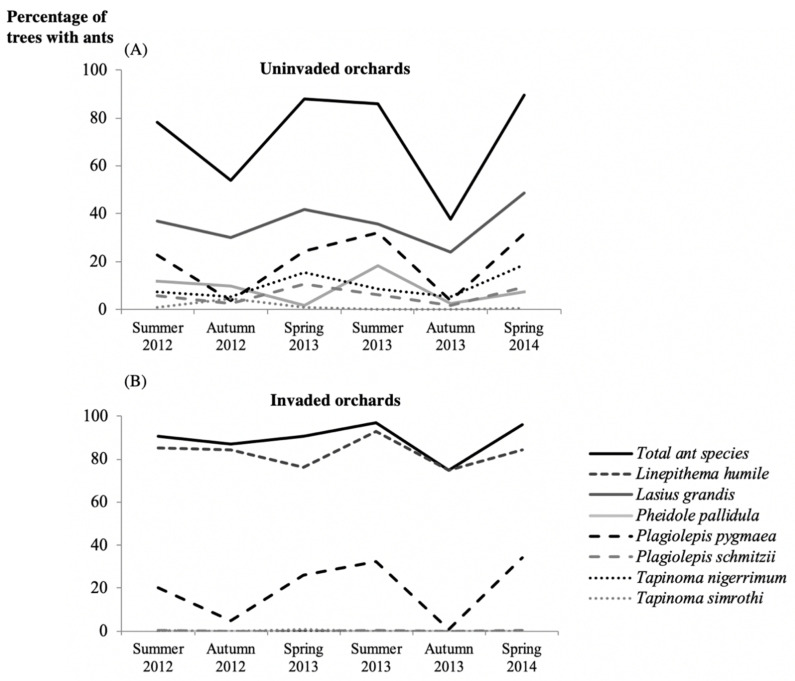
Seasonal variation in the percentage of trees with the most frequent ant species by season in the uninvaded (**A**) and invaded orchards (**B**). In each season, 240 trees were observed by modality.

Overall, ant frequency showed a seasonal pattern in both invaded and uninvaded orchards, with a maximum in spring/summer and a minimum in autumn (Figure 4). Still, the variation among seasons was more evident in non-invaded orchards, compared to those in which *L. humile* was present. The seasonal pattern also registered some variation in function of ant species. The level of variation among seasons was apparently lower for the most dominant species in each treatment, i.e., *L. grandis* and *L. humile* in uninvaded and invaded orchards, respectively (Figure 4). In addition, the seasonal peaks of activity varied among ant species. *Lasius grandis*, *T. nigerrimum* and *P. schmitzii* were more frequent in the spring, while the frequency of *L. humile*, *P. pygmaea* and *P. pallidula* was highest in the summer and that of *T. simrothi* in autumn. *Plagiolepis pygmaea* exhibited the same seasonal pattern and similar levels of activity in both invaded and uninvaded orchards. 

**Figure 5:**
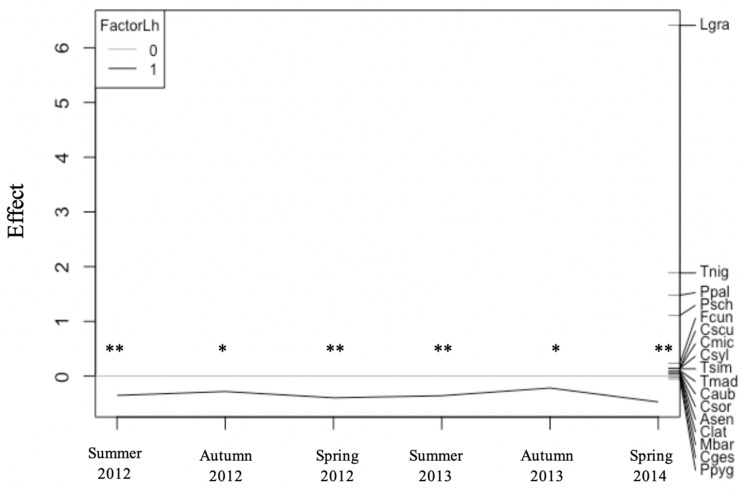
Principal response curves (PRC) representing the effects of the invasion by the Argentine ant on native ants foraging on citrus tree canopy. The left y axis represents deviances from the control (uninvaded orchards). Significant deviances based on Monte Carlo permutation tests are marked with one (*p* < 0.05) or two asterisks (*p* < 0.001). The right side of the figure represents ant species weight, accounting for the deviances of the PRC. The first axis explains 96% of the variance of species–environment. Legend: 0—native ant community (gray reference line); 1—ant community affected by the Argentine ant invasion (black line); Lgra—*Lasius grandis*, Tnig—*Tapinoma nigerrimum*, Ppal—*Pheidole pallidula*, Psch—*Plagiolepis schmitzii*, Fcun—*Formica cunicularia*, Cscu—*Crematogaster scutellaris*, Cmic—*Camponotus micans*, Csyl—*Camponotus sylvaticus*, Tsim—*Tapinoma simrothi*, Tmad—*Tapinoma madeirense*, Caub—*Crematogaster auberti*, Csor—*Crematogaster sordidula*, Asen—*Aphaenogaster senilis*, Clat—*Camponotus lateralis*, Mbar—*Messor barbarus*, Cges—*Camponotus gestroi*, Ppyg—*Plagiolepis pygmaea*.

PRC analysis revealed that the occurrence of native ants in uninvaded orchards was significantly higher (*p* = 0.001) than in the orchards in which the Argentine ant was present, in all sampling dates, during the two years´ study (Figure 5). Still, differences were higher in spring and summer than in autumn. *Lasius grandis* was the major contributor to the differences between invaded and uninvaded orchards, followed by *T. nigerrimum*, *P. pallidula*, and *P. schmitzii*.

## 4. Discussion

The evidence that invasive ants often become highly abundant in their introduced range and can outnumber native ants [4] is consistent with our results. We showed that the Argentine ant has a negative impact on the native ant community structure and that this effect is more or less pronounced depending on the season period. In addition, community processes are also likely to be affected by the Argentine ant invasion. The direct impacts on native ants modify networks and indirectly affect a variety of regulating and supporting services, disrupting ecosystem processes, such as trophic-based interactions, often leading to pest outbreaks [49]. We will thus focus our discussion on Argentine ants’ impacts on community structure (e.g., ant diversity and frequency), seasonal dynamics, and possible implications for citrus pest management.

### 4.1. Ant Community Structure

Ant communities of Mediterranean citrus orchards have been studied by several authors [18,20,21,41,50,51,52,53,54]. However, almost no studies addressed the impact of invasive species on the native ant community in this agroecosystem. Our results evidenced a negative impact of the Argentine ant on the native ant assemblages foraging on citrus canopy. Similar negative impacts have been reported in natural and forest ecosystems [6,8,17,55]. 

In invaded areas, the abundance of native ants can be reduced by over 90% ([4] and references therein) In our study, the orchards invaded by the Argentine ant showed a 44% and 76% reduction in the number and frequency of native ant species, respectively, compared to uninvaded orchards. A 60% decrease in native ant’s biodiversity was also reported by Menke et al. [17], in California riparian woodlands.

Overall, the uninvaded orchards showed a far more complex and richer ant community, composed of 16 native ant species, mainly dominated by *L. grandis*, *T. nigerrimum* and *P. pallidula* (72%). On the other hand, in invaded orchards, in which the ant community was dominated by the Argentine ant (80%), the native ant community was poorer and highly modified, limited to few species. As a result, mean ant species richness per orchard was lower in invaded orchards, compared to uninvaded ones. This pattern supports the ‘dominance–impoverishment rule’, according to which ant communities dominated by behaviourally dominant species are associated with low ant species richness [7,56]. Recently, Arnan et al. [56] suggested that this rule only applies to invaded communities, and not to native ones. 

Although invasive ants displace many native ant species, some are often able to persist ([4] and references therein, [55]). This is apparently the case of *P. pygmaea*, the only native ant species not affected by the presence of the Argentine ant. In our study, this species occurred in about 92% of the sampled orchards, with a similar frequency and seasonal pattern, in both invaded and uninvaded orchards. Similar observations were reported in other studies for *P. pygmaea* [57,58], and another species of the same genus, *P. schmitzii* [20]. *Plagiolepis pygmaea* is a common and not aggressive small species, which is tolerated by other ants, due to its submissive behaviour, allowing the coexistence with highly dominant species, such as *L. humile* [21,43,57]. In fact, species that coexist with *L. humile* are apparently small-sized species that can go unnoticed [6,55].

Invasive ants also compete with native ants indirectly via exploitative competition [4]. The native dominant species *L. grandis* and *P. pallidula* were only observed in the uninvaded orchards, and *T. nigerrimum* was only detected episodically in one of the invaded orchards. These results may support the hypothesis that the absence of the Argentine ant from citrus orchards in some areas of Algarve is, at least in part, related to interspecific competition with dominant native ant species. They may resist the invasion by the Argentine ant and limit its dispersion within the region. Competitive mechanisms underlying the displacement of native species by invasive ants may involve colony-level battles including the use of physical aggression by workers and nest raiding [4,59,60]. Colonies of *L. humile*, *L. grandis* and *T. nigerrimum* engage in aggressive and deadly battles (V. Zina, unpublished observation). Based on laboratory experiments, both *L. grandis* and *T. nigerrimum* showed to be strong competitors of *L. humile* [61] (V. Zina, unpublished observation). Other studies suggested that these dominant native species are able to resist and prevent the spread of the Argentine ant when its population is at low densities and/or the abiotic conditions are unfavourable to the invasion ([62] and references therein). Nevertheless, the outcome of the interaction between the Argentine and native dominant ants may be influenced by other factors, such as favourable habitat conditions and food resources availability [63,64]. Additional experimental data are needed, such as to test competition exclusion hypothesis, to confirm the hypothesis of biotic resistance.

It is known that the Argentine ant is able to secure the majority of food resources in areas where it meets native ants [60]. Additionally, the access to a carbohydrate-rich diet, such as hemipteran honeydew may allow invasive ants to feed workers at a high rate, making possible the maintenance of high dynamic densities, the defence of absolute territories, and the further monopolization of resources [65]. 

Our work has been carefully designed on the basis of an extensive, replicated approach to produce reliable ecological information associated with ant invasions of citrus ecosystems. Nevertheless, as the sampling was focused on ant species foraging on tree canopy, we may have underestimated possible interactions with other ant species, such as hypogaetic ants (e.g., *Solenopsis* spp., *Hypoponera* spp.). However, it is known that the Argentine ant has a small impact on those species [6,17,66]. Moreover, we were particularly interested in understanding the possible interactions with ant species that may establish trophic relationships with citrus insect pests, which are expected to be limited to citrus canopy.

### 4.2. Seasonal Patterns

The impact of the Argentine ant on the native ant community in citrus orchards was shown to be seasonally dependent, with stronger differences between invaded and uninvaded orchards, and in spring and summer, in comparison with autumn. A seasonal effect was also reported by other authors [35,67]. However, a different pattern to that registered by us was reported by Heller et al. [35]. These authors observed that the impact of the Argentine ant on native ants in a Northern California reserve was greater in the autumn than in the spring, and that invasive and native ants overlapped more often in the spring. Apparent seasonal differences in the impact of the Argentine ant may be related with differences in the seasonal dynamics of food resources, such as hemipteran honeydew, which is habitat dependent. For example, a high diversity of hemipteran species excreting honeydew is commonly associated with citrus crops, including aphids, whiteflies and scale insects, and the population density of these insect pests is maximal in spring and summer [20]. Therefore, it is expected that the abundance of a carbohydrate-rich diet for the Argentine ant will be higher in those seasons, in the case of citrus, but not necessarily in other habitats, with a low diversity and abundance of hemipteran species. 

Both the population dynamics of invasive and native species are expected to vary over time [68]. We found that season had a significant influence on the distributions of all the ants present in the orchards. However, this seasonal pattern registered some variation in their frequency depending on the ant species. For example, the dominant native species *L. grandis* established itself early in the year, reaching their peaks in the spring. In contrast, the Argentine ant showed the highest frequency in the summer. These different seasonal patterns are possibly related to the seasonal abundance of their preferred honeydew sources present in citrus canopy. In fact, *L. grandis* is known as an aphid/whitefly-tender species (e.g., *Aphis spiraecola* Patch, *Aleurothrixus floccosus* (Maskell)) [20,21,69], that are more abundant in springtime in parallel with citrus flushing, whereas the Argentine ant is more commonly associated with mealybugs and coccids (e.g., *Planococcus citri* (Risso), *Coccus hesperidum* Linnaeus) [21], with a population build-up occurring in the summer. 

Invasion impacts can increase or decrease in magnitude over time [17]. The Argentine ant exhibits differences in population density along the year. The abundance of the Argentine ant in summer is related to the increase in the spatial extent of the colony, with many small dispersed nests, expanding the foraging range, while in autumn the ants return to the old winter nests and the spatial extent of the colony contracts [35,70]. Then, they move from nest aggregation in the winter (colony contraction), to nest dispersion in summer (colony expansion). It has been suggested that in the summer, when the Argentine ant is more dispersed and active, encounters with native ants will be less frequent [35]. However, with their peak abundance in the summer, we believe that major ecological impacts are expected to occur. In our work, Argentine ants’ invasion varied in magnitude among orchards and consequently in the impact on native ant species. A negative correlation was significant in the summer and spring but not in the autumn. Menke et al. [17] suggested that variation in invader’s abundance may be due to site-specific factors. In citrus orchards, it is likely to depend on the nutritional needs and resources abundance, yet a consequence of the season [64].

### 4.3. Implications for Citrus Pest Management

Some ant species are considered citrus pests [30], because they may originate direct damage on citrus plants, by feeding on leaves, shoots and buds, such as *T. nigerrimum* [71]. More frequently, ants’ pest status on citrus is due to their mutualistic relationship with honeydew-producing hemipterans and consequent negative impact on the natural enemies of these sap-sucking insect pests. The disruption of biological control of hemipteran insect pests by ants has been reported by different authors [20,29,72,73]. The impact of ants in the levels of parasitism and predation of citrus pests is apparently dependent on ant species and population density [54,74,75].

The changes in native fauna driven by invasive species can have cascading consequences on the ecosystems services they provide [49]. Invasive and native ant species may preferentially explore different sources of honeydew. For example, Zina et al. [21] observed a positive correlation between Argentine ant and mealybugs and other scales insects (e.g., *C. hesperidum*), as well as between *L. grandis* and aphids and whiteflies, and between *T. nigerrimum* and *I. purchasi*. Different associations were reported by other authors, such as *P. pallidula* and *L. grandis* with mealybugs [20]. Such interactions among different honeydew-producing hemipterans may be of general importance for the Argentine ant’s successful invasion in citrus orchards. There is a need to better understand the preferential associations between ants and honeydew-producers on citrus. Considering that ants may disrupt the biological control of citrus pests and that they may differ in their preferences for foraging in different honeydew sources (e.g., whiteflies, mealybugs, aphids) in the citrus canopy, we may expect that the different composition of ant assemblages in a citrus orchard will represent a different risk of insect pest outbreaks. For example, we would expect that the presence of the Argentine ant will favour the population build up and will increase the risk of outbreaks of the citrus mealybug, *P. citri*, a major pest of citrus crops in the Mediterranean area [30]. Besides differences in honeydew preference, different aggressive behaviour and potential for disrupting biological control, as well as different ability to build up large populations among ant species may also influence pest outbreak risks. Furthermore, ants have been recently reported as possible vectors of citrus diseases [76], and this role may also differ among ant species. Therefore, knowledge on the structure and composition of ant communities in citrus orchards, and the influence of invasive alien species, such as the Argentine ant, on native ant species, is of relevance for decision making in citrus pest management. Novel approaches of effective and selective ant control (e.g., prey-baiting [77]) may contribute to improve the biological control of citrus pests.

## 5. Conclusions

The results obtained in the present study provide strong evidence that the structure and composition of the native ant community foraging on the citrus canopy are affected by the presence of the Argentine ant, with a significant reduction in the diversity and frequency of native ant species in invaded orchards. The level of impact of the Argentine ant was season dependent, with a higher impact registered in spring and summer, in comparison with autumn, supporting our hypothesis that the dominance of the Argentine ant is expected to be higher in warmer periods of the year. 

Our data also support the hypothesis that the interspecific competition with dominant native ant species, such as *L. grandis*, *P. pallidula* and *T. nigerrimum* may prevent the invasion by the Argentine ant in certain areas. However, the interaction between the Argentine and dominant ant species may be influenced by other factors, such as habitat conditions and food resources.

Future studies should aim at uncovering the mechanisms underlying the impacts of Argentine ant invasion in citrus orchards, namely through competitive interactions with dominant native species in the initial stages of invasion, and its associations with honeydew-producing hemipterans.

## Figures and Tables

**Table 1:** Ant species collected in Argentine ant invaded and uninvaded citrus orchards of Algarve, Portugal.

Subfamily	Number of Specimens				Number of Orchards in Which Each Ant Species Was Found			
	Uninvaded Orchards (N = 12)		Invaded Orchards (N = 12)		Uninvaded Orchards (N = 12)		Invaded Orchards (N = 12)	
Ant Species								
	N	%	N	%	N	%	N	%
Dolichoderinae								
*Linepithema humile*	0	0	5153	86.37	0	0	12	100
*Tapinoma madeirense*	22	0.44	0	0	2	16.67	0	0
*Tapinoma nigerrimum*	835	16.82	2	0.03	8	66.67	1	8.33
*Tapinoma simrothi*	32	0.64	7	0.12	6	50.00	1	8.33
Formicinae								
*Camponotus gestroi*	0	0	1	0.02	0	0	1	8.33
*Camponotus lateralis*	6	0.12	0	0	1	8.33	0	0
*Camponotus micans*	25	0.50	0	0	1	8.33	0	0
*Camponotus sylvaticus*	12	0.24	3	0.05	4	33.33	1	8.33
*Formica cunicularia*	24	0.48	0	0	2	16.67	0	0
*Lasius grandis*	2099	42.28	0	0	10	83.33	0	0
*Plagiolepis pygmaea*	1159	23.35	783	13.12	10	83.33	12	100
*Plagiolepis schmitzii*	224	4.51	3	0.05	7	58.33	2	16.67
Myrmicinae								
*Aphaenogaster senilis*	4	0.08	0	0	2	16.67	0	0
*Crematogaster auberti*	41	0.83	4	0.07	2	16.67	1	8.33
*Crematogaster scutellaris*	33	0.66	1	0.02	4	33.33	1	8.33
*Crematogaster sordidula*	56	1.13	9	0.15	3	25.00	1	8.33
*Messor barbarus*	4	0.08	0	0	3	25.00	0	0
*Pheidole pallidula*	388	7.82	0	0	8	66.67	0	0
Total Number of Specimens (*N*)	4964		5966					
Species Richness (*S*)	16		10					

**Figure A1:**
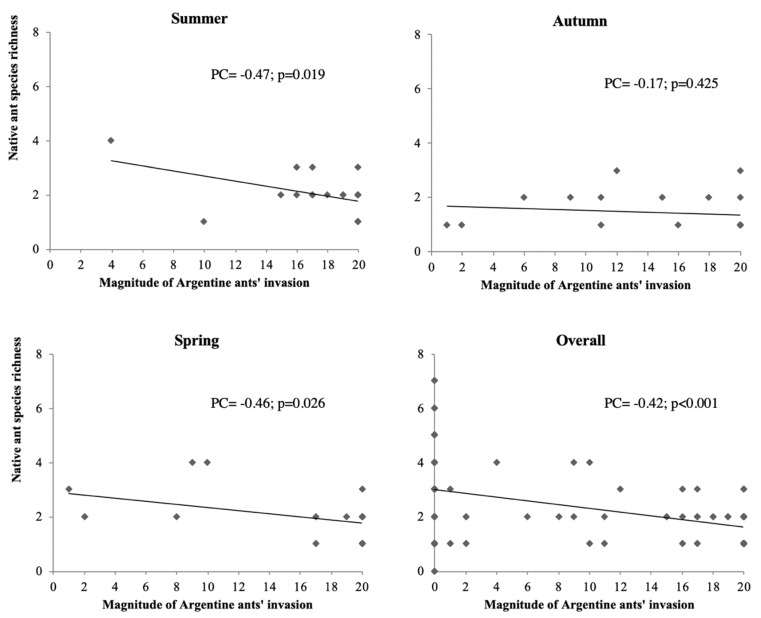
Scatterplot displaying a negative correlation between the magnitude of invasion, i.e., the percentage of infested trees by the Argentine ant, and the number of native ant species per orchard at summer, autumn, spring and overall. Pearson’s Correlation (PC) and *p*-value are shown.

**Figure A2:**
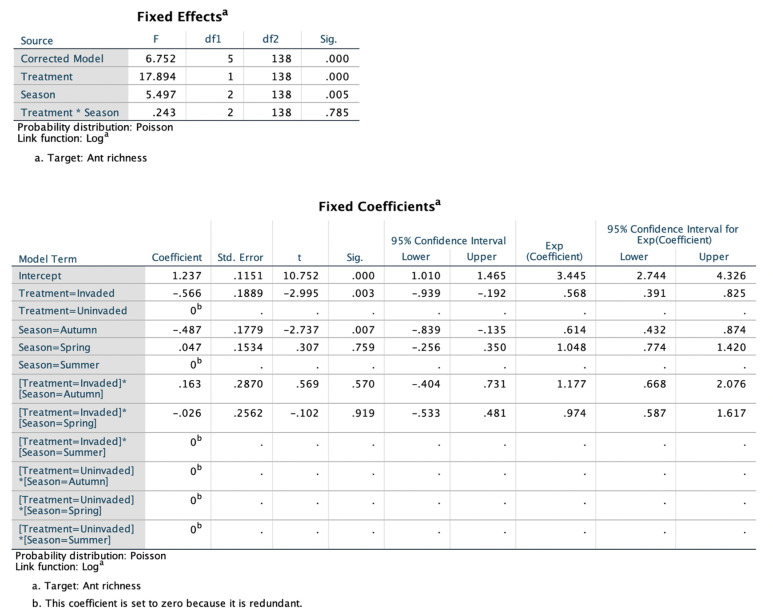
Results for the generalized linear mixed model (GLMM) of ant richness fitted by the Poisson distribution, with treatment, season and their interaction as fixed factors, the orchards as subjects with repeated measures and the year as a random variable.

**Figure A3:**
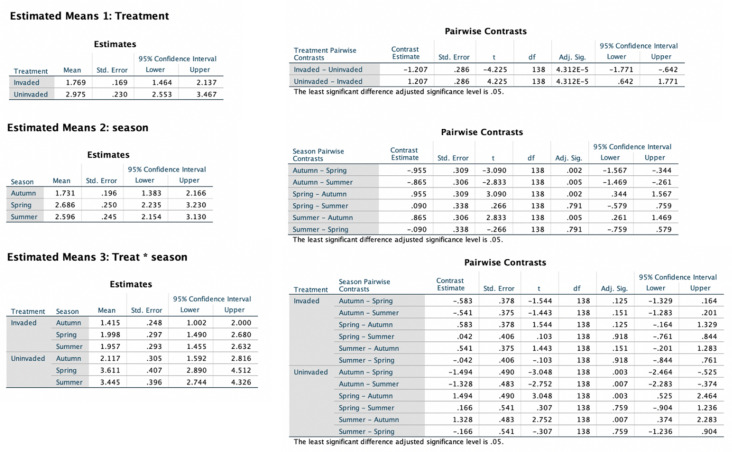
Results for the GLMM’s estimated marginal means, showing the pairwise combinations of treatments (invaded/uninvaded orchards) and seasons (summer, autumn, spring).
